# A Case of Chronic Recurrent Multifocal Osteomyelitis (CRMO)

**DOI:** 10.7759/cureus.72058

**Published:** 2024-10-21

**Authors:** Anjali Alamshaw, Lori Zadoorian, Makayla Carlson, Fidel Perez, Morgan Green

**Affiliations:** 1 Pediatrics, Western University of Health Sciences, Pomona, USA; 2 Pediatrics, Loma Linda University Medical Center, Loma Linda, USA; 3 Pediatrics, Riverside University of Health Sciences, Moreno Valley, USA

**Keywords:** chronic recurrent multifocal osteomyelitis (crmo), chronic recurrent non-bacterial osteomyelitis (cno), osteomyelitis, pediatric chronic osteomyelitis, recurrent osteomyelitis

## Abstract

Chronic recurrent multifocal osteomyelitis (CRMO) is a rare but underdiagnosed, severe inflammatory bone disease that primarily affects children. It’s typically characterized by bone pain, especially within the metaphysis of long bones, clavicles, and vertebrae, but it can present in any bone and with varied presentation, including extraosseous symptoms that may be associated with other autoimmune/inflammatory diseases. Chronic recurrent multifocal osteomyelitis is a sterile bone disease that does not typically have an infectious source. Its pathogenesis involves the dysregulation of the innate immune system via upregulation of cytokines, inflammasomes, and osteoclastic properties. Diagnostic testing is nonspecific and includes X-ray and MRI, bone biopsy, inflammatory markers, and autoimmune markers. Chronic recurrent multifocal osteomyelitis is established by the diagnosis of exclusion. First-line treatment is nonsteroidal anti-inflammatory drugs (NSAIDs); if vertebral involvement is present, a bisphosphonate is typically added. This case report emphasizes the importance of considering CRMO as a differential diagnosis of multifocal bone pain in children.

## Introduction

Chronic recurrent multifocal osteomyelitis (CRMO) is a rare, noninfectious autoinflammatory disorder that primarily affects children and adolescents. The exact pathogenesis of CRMO is unclear; however, studies suggest that there is dysregulation of the innate immune system’s cytokines and interleukins (ILs), resulting in downregulated anti-inflammatory ILs, IL-10, and IL-19, resulting in a pro-inflammatory state. This ultimately causes the promotion of osteoclastic bone resorption and the resulting bone pain and skeletal findings on workup [1]. 

The incidence of CRMO has been reported to be four per million children [2]. However, due to underdiagnosis in clinical practice, it is difficult to assess the accurate incidence and prevalence of CRMO. Thus, it is believed that CMRO may not be as rare as it is assumed. There is a higher incidence in females (2:1) compared to males and Caucasians compared to other races [2].

Symptoms secondary to CRMO typically develop in children between the ages of seven and nine years old and include the gradual onset of pain and edema over the bone, primarily in the metaphysis of long bones, clavicle, and vertebrae. The pain is worse at night and may be accompanied by fevers [3]. In addition, the patient may present with other extraosseous symptoms that may include dermatologic manifestations, such as psoriasis-like or pustular rash, intermittent fevers, or gastrointestinal manifestations that resemble Crohn’s disease, which may indicate comorbidity with other autoimmune/inflammatory diseases, such as ankylosing spondylitis, psoriasis, or inflammatory bowel disease [1]. 

Chronic recurrent multifocal osteomyelitis typically has no growth on bone biopsy, which can rule out malignancy and infection [4]. Whole-body MRI is typically utilized as the imaging modality for diagnosing and monitoring the disease progression of CRMO, which will typically present with lytic or sclerotic bone lesions [5]. Lab work may show a nonspecific but moderately elevated C-reactive protein (CRP) and erythrocyte sedimentation rate (ESR). 

First-line treatment of CRMO without vertebral involvement is nonsteroidal anti-inflammatory drugs. When vertebral involvement is identified, bisphosphonates have been utilized. Other treatments that have been used include tumor necrosis factor-alpha (TNFα) antagonists, steroids, methotrexate, sulfasalazine, colchicine, and azithromycin. There is current discussion regarding the efficacy of IL-1 antagonists [3].

## Case presentation

Our patient was a seven-year-old male who was born at thirty-four weeks with a history of attention deficit hyperactivity disorder (ADHD) (on lisdexamfetamine), autism, and one febrile seizure and who presented to the emergency department with bilateral hip pain. His parents did not recall any precipitating events and recalled that the patient often woke in the middle of the night groaning with pain. The patient was previously able to run around with no issues at baseline. There is no family history of musculoskeletal disorders. At an outside hospital, X-rays of the left hip, pelvis, and left femur were unremarkable. The patient was admitted due to elevated ESR (59 mm/hr) and CRP (12.7 mg/L) at the outside hospital. 

On admission, the patient’s physical exam was notable for pain upon hip flexion and internal rotation, positive left Trendelenburg sign, and antalgic gait with left-sided limp, but otherwise benign exam with full passive range of motion in hip flexion/extension and internal/external rotation. He was afebrile upon admission.

**Figure 1:**
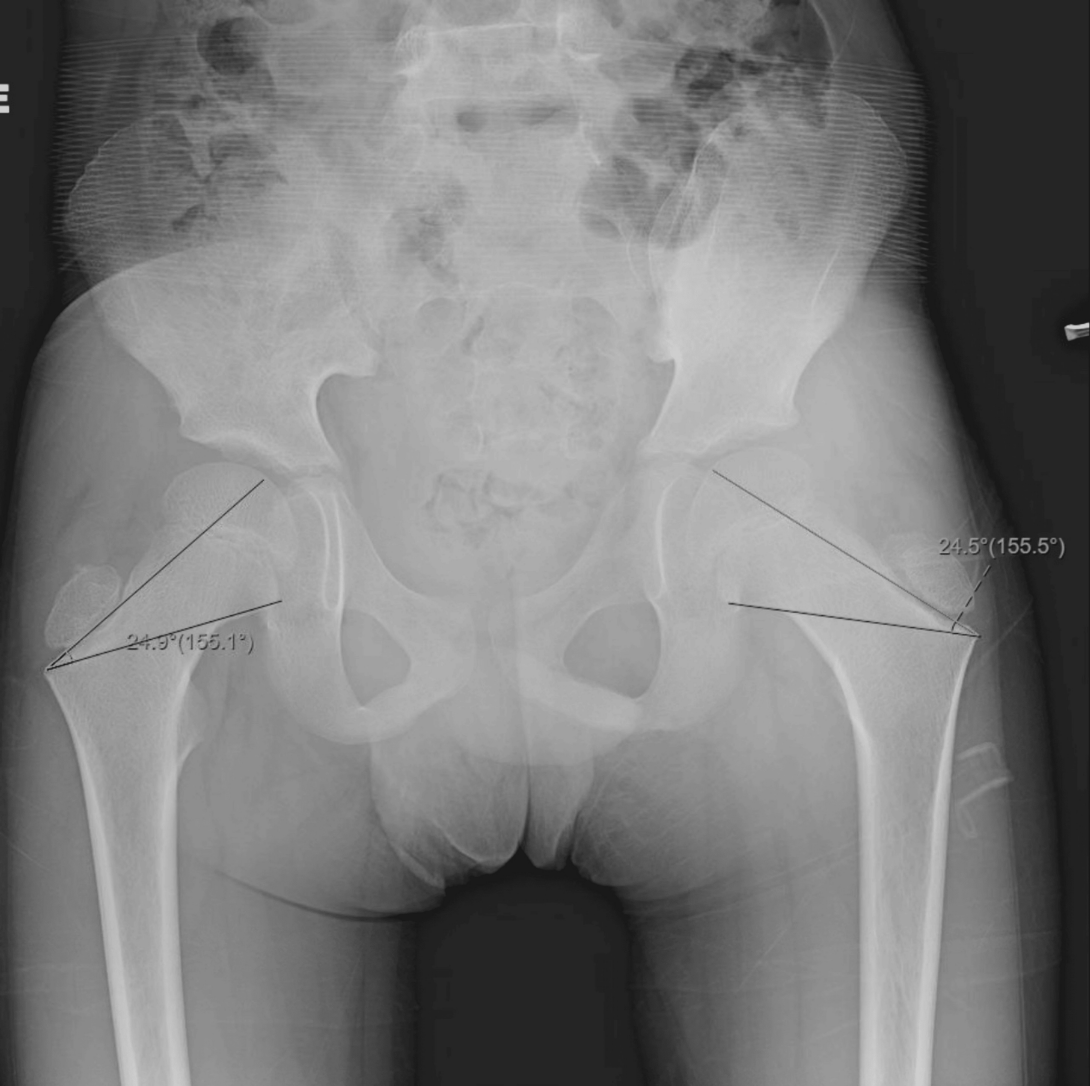
Hip X-ray showing asymmetric prominence of the left greater trochanter physis

**Figure 2:**
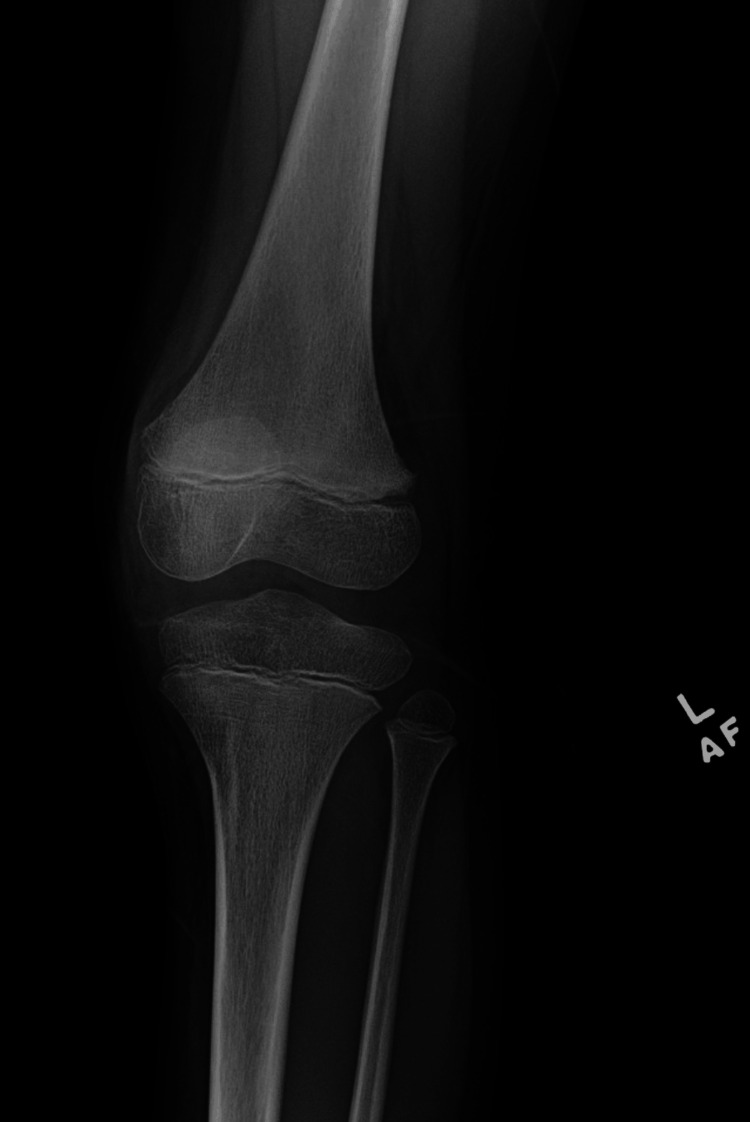
X-ray of the left knee showing soft tissue swelling of the medial knee

**Figure 3:**
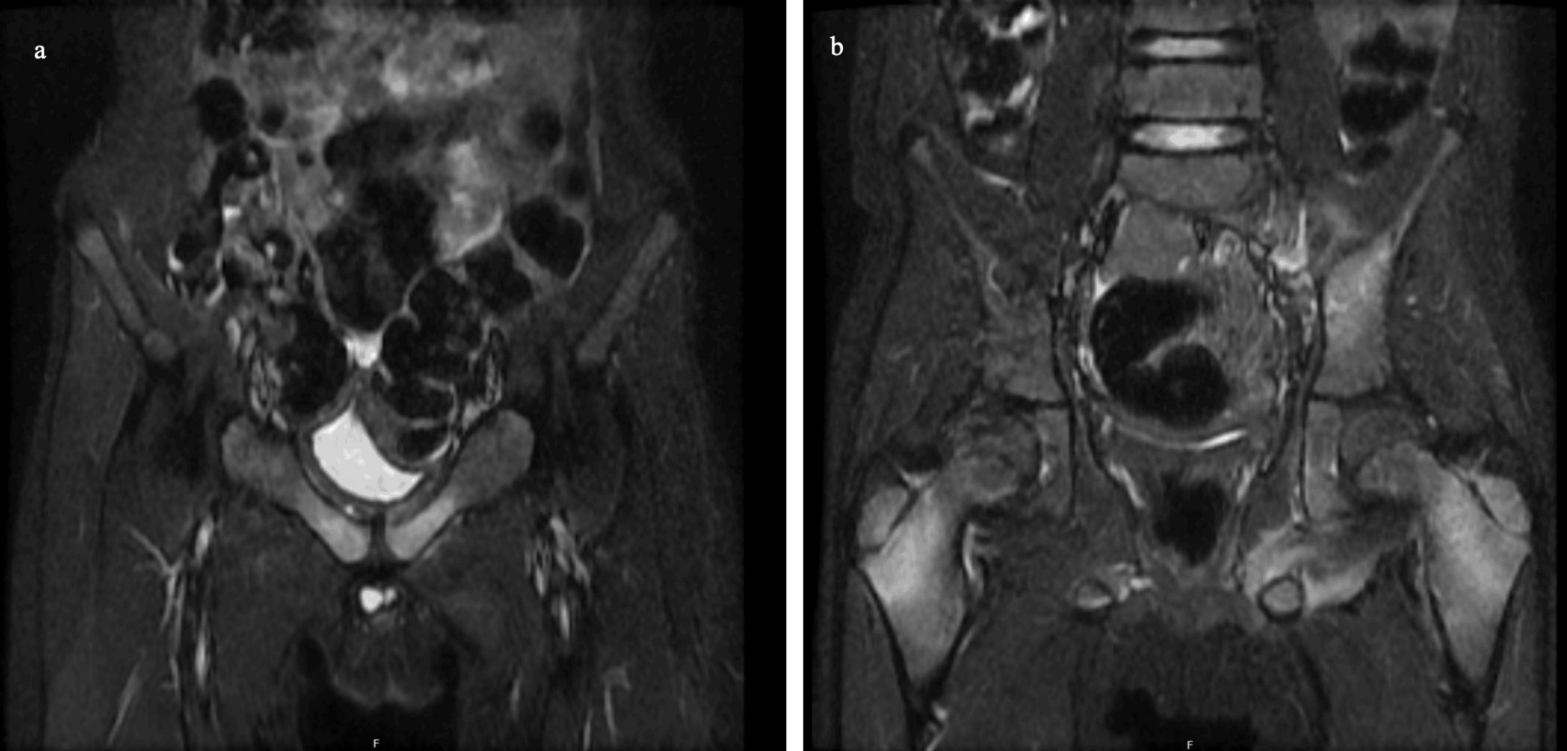
Pelvic MRI showing erosive findings at the left sacroiliac joint, asymmetric bone marrow edema at the bilateral pubic rami, and greater trochanteric apophysis/periphyseal region with adjacent intramuscular edema and periostitis

During the patient’s hospital stay, he had one recorded fever of 100.5°F. Laboratory results indicated no leukocytosis, unremarkable creatine kinase (CK) (51 U/L), and unremarkable lactate dehydrogenase (LDH) (199 U/L). The ESR and CRP were elevated at 60 mm/hr and 0.94 mg/L, respectively. Throughout his 14-day hospital course, the ESR and CRP slowly declined. Blood cultures demonstrated no growth. Hip X-ray was remarkable for asymmetric prominence of the left greater trochanter physis (Figure 1), and left knee X-ray demonstrated soft tissue swelling of the medial knee (Figure 2). An MRI of the pelvis demonstrated erosive findings at the left sacroiliac joint as well as asymmetric bone marrow edema at the bilateral pubic rami and greater trochanteric apophysis/periphyseal region with adjacent intramuscular edema and periostitis (Figure 3).

Due to suspicion for osteomyelitis, particularly due to erosive sacroiliac findings on MRI, the patient was started on a course of IV cefazolin. There was lower concern at the time for other differential diagnoses, including multifocal septic arthritis, juvenile idiopathic arthritis, and Legg-Calve-Perthes. Following these focal imaging results, the patient underwent a left iliac bone core biopsy, which showed trabecular bone with bland hypocellular marrow but no evidence of malignancy or osteomyelitis. Tissue and anaerobic cultures from biopsy specimens showed no growth. After consulting with the pediatric rheumatology, hematology/oncology, and infectious diseases team, the likely differential was CRMO rather than osteomyelitis, with the patient meeting Bristol diagnostic criteria. The patient was transferred to a center that offers whole-body MRI, which he underwent later in his disease course. 

**Figure 4:**
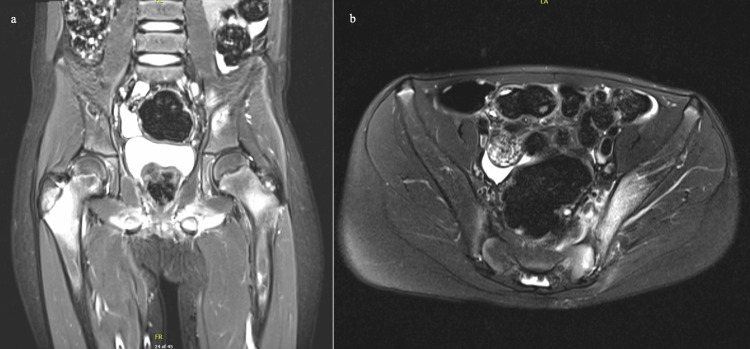
MRI images showing edema-like marrow throughout pelvis and femur

HLA-B27 was collected and later found to be positive. An MRI of the pelvis and hip showed edema-like marrow signals throughout the pelvis and lower extremity bones, including the tibias and femurs (Figure 4). Naproxen was given for pain relief. The patient was discharged with cephalexin 800 mg every eight hours (q8hr) for a six-week course, which was chosen since the patient was on cefazolin during hospitalization. At a follow-up visit, the patient’s mother noted that the patient had returned to baseline and had been compliant with medication. 

**Figure 5:**
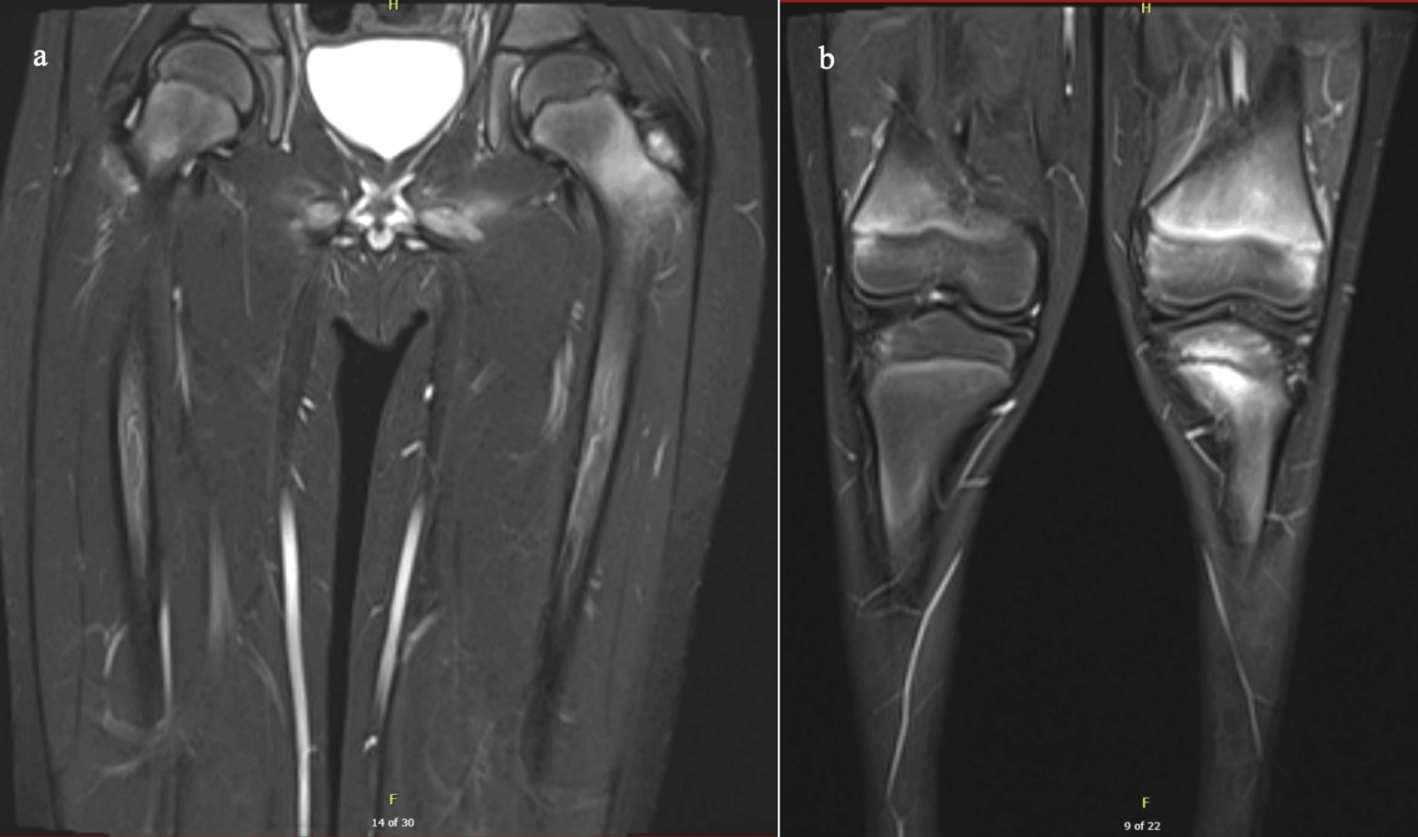
Whole body MRI showing edema-like marrow signal noted throughout the tibias and femurs

Shortly after, the patient was readmitted for fevers. Laboratory results showed elevated CRP (3.2 mg/L), leukocytosis (14.8 x 103 white blood cells/µL), and thrombocytosis (587 x 109 platelets/L). The patient was empirically started on IV vancomycin after discussion with the infectious diseases team in response to new-onset fevers and elevated inflammatory markers, which was discontinued upon negative blood cultures, indicating a more likely autoimmune rather than infectious etiology. A whole-body MRI was completed inpatient to rule out new concerns, which showed an edema-like marrow signal noted throughout the pelvis and lower extremity bones, including the tibias and femurs (Figure 5). The patient was scheduled with a follow-up rheumatology outpatient appointment upon discharge, with recommendations from their team to take a one-time dose of 250 mg IV methylprednisolone inpatient and continue naproxen 250 mg two times a day (BID) at home. Methylprednisolone was started after infection was ruled out and the patient was found to be HLA-B27 positive, qualifying for enesthesitis-related arthritis. The patient was also set up with follow-up appointments with the infectious diseases and orthopedic surgery teams.

During the patient’s first rheumatology follow-up appointment, the patient’s mom noted that the patient had been adherent to daily naproxen and that the patient had had some “funny” walking with no new joint swelling. Due to previously noted positive HLA-B27 status and sacroiliitis on MRI, the rheumatologist diagnosed the patient with enthesitis-related arthritis, which may be a CRMO-related association [5]. At another rheumatology follow-up appointment six months later, the patient reported improved back pain on his current naproxen regimen, which was continued after the appointment. The patient was scheduled for another outpatient visit a few months later to repeat an MRI. His infectious disease and orthopedic surgery appointments were consistent with his rheumatology visits. The patient will continue follow-up appointments with rheumatology and daily naproxen.

## Discussion

Chronic recurrent multifocal osteomyelitis is an autoinflammatory bone disease that typically occurs in children. Chronic recurrent multifocal osteomyelitis can occur in any bone; however, it frequently involves the metaphysis of long bones, especially the femoral and tibial metaphysis, the pelvis, the spine, the clavicle, and the mandible [6]. Our case involved a seven-year-old patient with hip pain that was initially localized to the left hip and then, one month later, was localized to the right hip. Our patient initially presented with symptoms localized to a single site, but multiple sites were later identified on imaging, which is consistent with the trajectory of a majority of CRMO patients [7]. 

The etiology of CRMO is unclear and is considered to be an autoimmune inflammatory condition [2]. Chronic recurrent multifocal osteomyelitis can be associated with other inflammatory conditions, such as psoriasis and inflammatory bowel disease (IBD), and it may evolve into spondyloarthropathy [8]. Our patient did not have any individual or family history of autoimmune diseases.

The pathophysiology of CRMO is not fully understood. It may involve an imbalance in cytokine expression that leads to a pro-inflammatory state contributing to bone inflammation. Reduced levels of regulatory cytokines IL-10 and IL-19 may occur in the context of increased expression of pro-inflammatory cytokines (such as IL-6) and chemokines (such as IL-8) [9, 10]. Decreased IL-10 levels may contribute to osteoclastogenesis by promoting increased inflammasome activation. Increased levels of pro-inflammatory cytokines can also promote osteoclastogenesis [11]. There are various mechanisms of study, but one suggests that pro-inflammatory cytokines upregulate RANKL in osteoblasts, which can then bind to its corresponding RANK receptor and induce osteoclastogenic signals [12].

In diagnosing CRMO, multiple factors can be considered, such as age, clinical presentation, and a positive HLA-B27 test. CRMO is ultimately a diagnosis of exclusion. Chronic recurrent multifocal osteomyelitis can be differentiated from bacterial osteomyelitis (BOM) based on clinical presentation, as laboratory studies are often similar [13]. Laboratory findings often reveal increased levels of inflammatory markers like ESR, CRP, and leukocytosis [14]. Children with BOM more frequently presented with local inflammatory signs, fever, and abscesses compared to children with CRMO. Our patient had one fever of 100.5°F, but he had fewer systemic symptoms than would have been expected with infection, and there was no leukocytosis on lab findings. Our patient’s elevated ESR and CRP levels are consistent with the inflammatory nature of CRMO. Additionally, our patient tested positive for HLA-B27 and received a diagnosis of enthesitis-related arthritis in addition to the CRMO diagnosis, which discloses a possible overlap between CRMO and other autoimmune conditions [5].

Magnetic resonance imaging is highly sensitive for detecting inflammatory lesions in CRMO and can also exclude alternative diagnoses such as bacterial infection [15]. Whole-body MRI can reveal multifocal disease and patterns of skeletal involvement, which are typically characteristic of CRMO. Specific skeletal sites may be involved, such as the tibia and femur. Clavicular involvement can be involved in CRMO lesions as well and can indicate a more probable diagnosis of CRMO rather than BOM [16]. Although individual lesions aren’t specific to a CRMO diagnosis, certain patterns, such as bilateral tibial disease, may be found involving skeletal sites [16]. Long bone metaphysis site lesions in children are typically characteristic of CRMO [17]. If clinical presentation and whole-body MRI findings align well, then there may not be a need for a biopsy. However, a biopsy can also be used for diagnosis in cases that don’t align with typical diagnostic criteria of CRMO; CRMO biopsy findings typically show inflammatory changes with no bacterial growth [18]. Our patient’s left iliac bone core biopsy showed no growth or evidence of osteomyelitis. Our patient had erosive findings at the left sacroiliac joint that were revealed on MRI and nonspecific findings on whole-body MRI (edema-like marrow signal throughout the pelvis and lower extremity bones, including the tibias and femurs) that, along with his clinical presentation, likely indicated a diagnosis of CRMO.

The first-line treatment of CRMO without spinal involvement is nonsteroidal anti-inflammatory drug (NSAID) use, which inhibits osteoclastic activity. Tumor necrosis factor-alpha inhibitors and bisphosphonates can also be effective for symptomatic relief and organization of bone structure [19]. Bisphosphonates can be primarily useful when vertebral involvement is notable in work-up. Interleukin-1 antagonists may be efficacious as well, but further studies need to be conducted [20]. Corticosteroids may also be effective in CRMO patients, as they negatively affect pro-inflammatory cytokine expression [2]. Our patient was treated with a one-time dose of methylprednisolone and naproxen twice daily. Long-term management of CRMO also includes routine monitoring through repeat lab tests and MRIs and follow-up appointments with rheumatology, which are consistent with the care that our patient has received [11].

## Conclusions

Chronic recurrent multifocal osteomyelitis is an autoinflammatory bone disorder that should be considered as a differential diagnosis in children presenting with multifocal bone pain. Chronic recurrent multifocal osteomyelitis is a diagnosis of exclusion with modalities such as whole-body MRI and biopsy contributing to a diagnosis of CRMO. This case report demonstrates the variability throughout the disease course of CRMO as well as the necessity of multi-disciplinary care involvement in order to properly diagnose, treat, and improve patient morbidity.
